# Validation of an algorithm to identify incident interstitial lung disease in patients with rheumatoid arthritis

**DOI:** 10.1186/s13075-021-02655-z

**Published:** 2022-01-03

**Authors:** M. Meehan, A. Shah, J. Lobo, J. Oates, C. Clinton, N. Annapureddy, F. Xie, J. Zhuo, M. I. Danila, B. R. England, J. R. Curtis

**Affiliations:** 1 University of Alabama at Birmingham, Division of Clinical Immunology and Rheumatology, AL Birmingham, USA; 2 Duke University, Durham, NC USA; 3 University of North Carolina at Chapel Hill, Chapel Hill, NC USA; 4 Medical University of South Carolina, Charleston, SC USA; 5 Vanderbilt University Medical Center, Nashville, TN USA; 6 Bristol Myers Squibb, New York, USA; 7 University of Nebraska Medical Center, Omaha, NE USA; 8 Veterans Affairs Nebraska-Western Iowa Health Care System, Omaha, NE USA

**Keywords:** Rheumatoid arthritis, Interstitial lung disease, Validation, Algorithm, Positive predictive value, Administrative data

## Abstract

**Background/purpose:**

Interstitial lung disease (ILD) is an important problem for patients with rheumatoid arthritis (RA). However, current approaches to ILD case finding in real-world data have been evaluated only in limited settings and identify only prevalent ILD and not new-onset disease. Our objective was to develop, refine, and validate a claims-based algorithm to identify both prevalent and incident ILD in RA patients compared to the gold standard of medical record review.

**Methods:**

We used administrative claims data 2006–2015 from Medicare to derive a cohort of RA patients. We then identified suspected ILD using variations of ILD algorithms to classify both prevalent and incident ILD based on features of the data that included hospitalization vs. outpatient setting, physician specialty, pulmonary-related diagnosis codes, and exclusions for potentially mimicking pulmonary conditions. Positive predictive values (PPV) of several ILD algorithm variants for both prevalent and incident ILD were evaluated.

**Results:**

We identified 234 linkable RA patients with sufficient data to evaluate for ILD. Overall, 108 (46.2%) of suspected cases were confirmed as ILD. Most cases (64%) were diagnosed in the outpatient setting. The best performing algorithm for prevalent ILD had a PPV of 77% (95% CI 67–84%) and for incident ILD was 96% (95% CI 85–100%).

**Conclusion:**

Case finding in administrative data for both prevalent and incident interstitial lung disease in RA patients is feasible and has reasonable accuracy to support population-based research and real-world evidence generation.

**Supplementary Information:**

The online version contains supplementary material available at 10.1186/s13075-021-02655-z.

## Introduction

Rheumatoid arthritis (RA) is associated with a variety of extra-articular pulmonary manifestations, the best characterized being interstitial lung disease (ILD). RA-ILD is a serious disease that is challenging to diagnose and treat and results in premature morbidity and mortality [1–3]. There is variation in the reported estimated prevalence and incidence of RA-ILD depending on the case definition used and the RA-related characteristics of the population studied. The reported prevalence of ILD in patients with RA ranges from 5 to 10%, with annualized incidence rates as high as 4.1 per 1000 people for clinically significant RA-ILD [4–7]. Accurately identifying RA patients with ILD within large patient cohorts and registries is important in order to support studies examining ILD-related epidemiology and long-term outcomes in this population, enable allocation of healthcare resources based on patient needs, and allow for efficient screening for the identification of those who might be eligible for interventional studies of therapies to treat ILD.

Case ascertainment of RA-ILD at a population level has been challenging, and algorithms using administrative databases for the identification of RA-ILD cohorts have previously been applied to large healthcare databases without validation [8–13]. To improve the validity of case identification, some approaches have used imaging reports to validate ILD claims diagnosis without additional medical record review [14]. As one advancement, a recent study using Veterans Affairs (VA) administrative data created and validated several variants of claims-based algorithms to identify prevalent RA-ILD compared to the gold standard of medical record review [15]. A second study, conducted in a single-state medical center network, validated an algorithm to identify prevalent RA-ILD in claims data and showed a PPV as high as 72% but did not evaluate incident ILD [16]. The generalizability of a prevalent ILD algorithm in more diverse environments is unknown.

A validated approach to find incident ILD is important given the interest in measuring disease incidence and in identifying newly diagnosed patients who might be referred for additional services (e.g., referral to a RA-ILD clinical trial). Therefore, the objectives of this study were to validate variants of previously developed RA-ILD definitions for prevalent RA-ILD in alternative administrative data sources to evaluate their generalizability, and as a novel feature of this work compared to past efforts, to extend these algorithms to allow for identification of incident RA-ILD. To accomplish these objectives, we linked administrative data from Medicare to electronic medical records, allowing for comparisons of identified cases from claims data to a gold standard of medical record review by ILD experts.

## Methods

### RA cohort identification

We identified and included RA patients treated with conventional, biologic, or synthetic disease-modifying anti-rheumatic drugs (DMARDs) from 01/01/2006 to 12/31/2015 using ICD-9-CM codes in Medicare data from 01/01/2006 through 09/30/2015, and ICD-10-CM codes from 10/1/2015 to 12/31/2015. To identify RA patients, we required two or more claims for RA (ICD-9-CM 714.0 or 714.2) occurring between seven and 365 days apart, with at least one from a rheumatologist. In addition, we required at least one prescription or infusion of an RA medication (hydroxychloroquine, sulfasalazine, methotrexate, leflunomide, infliximab, etanercept, adalimumab, golimumab, certolizumab pegol, abatacept, tocilizumab, rituximab, tofacitinib) [17]. To ensure an adequate baseline period that would allow the identification of incident ILD, we required greater than or equal to 12 months of continuous Medicare A+B-C coverage prior to the start of follow-up. The date of RA cohort eligibility (i.e., RA Cohort Index date) was defined as the date the patient met all three of the above requirements (RA diagnosis, DMARD, and at least 12 months of continuous coverage). The project was initiated in 2017, and given the availability of the Medicare claims data, we established a cohort inclusion cutoff date of 12/31/2015. All available Medicare data prior to the date of RA cohort eligibility were included in the baseline period.

We excluded patients with a diagnosis of other autoimmune diseases (e.g., systemic lupus erythematosus, scleroderma, myositis), malignancy except for non-melanoma skin cancer, HIV, or history of organ transplantation using all available data prior to the index date. All study activities were conducted in accordance with institutional review board approval at each medical center, and the data use was governed by a data use agreement from CMS.

### Case qualification definitions

Each case was classified as to the type of inpatient or outpatient visit associated with the first ILD diagnosis. Cases were characterized based on the place of service where the initial diagnosis appeared in the claims data using a “case qualifying” status as follows: HospitalPrimary = inpatient primary diagnosis, HospitalNonPrimary = inpatient non-primary diagnosis, OutpatientCT = outpatient diagnosis preceded by CT within 90 days, and OutpatientHospital = outpatient diagnosis preceded by hospitalization within 90 days.

### Medical record confirmation of ILD

The RA cohort of patients with suspected prevalent and incident ILD was further restricted to patients at five participating academic medical centers where linked medical records were available: Duke University, The Medical University of South Carolina (MUSC), The University of Alabama at Birmingham (UAB), The University of North Carolina (UNC), and Vanderbilt University Medical Center (VUMC). Each center cross-referenced the information available from the Medicare administrative data with the corresponding information in their center’s corresponding electronic medical records using either a search tool run against a central data warehouse or repository (e.g., i2b2) or their own local ILD registry. Medical record reviewers at each site abstracted clinical data, including clinical notes, date of diagnosis, CT scan reports, chest x-ray reports, lung pathology reports, and pulmonary function tests (PFTs), into a case report form. Case report data from all sites was de-identified, aggregated, and adjudicated independently by two ILD experts (pulmonology and rheumatology). The possible adjudication outcomes for each ILD case included: confirmed, not confirmed, insufficient information to determine, or not retrievable, with discordance in adjudication resolved by consensus (initial agreement as measured by kappa = 0.96, 95% CI 0.93–1.00). ‘Not confirmed’ indicated patients who had sufficient information by which to judge ILD case status, and the patient did not have ILD. “Insufficient information to determine” indicated suspected cases where there was insufficient data in the EHR to make a determination as to whether they had ILD or not (e.g., mention of an ILD-related diagnosis, but no primary data [e.g., HRCT results] was available). “Not retrievable” indicated that the EHR record could not be linked or obtained for the patient. Based on the entirety of the medical record, the adjudicators and site abstractors subsequently classified each confirmed case as incident ILD, prevalent ILD, or not able to be classified. The results of adjudicated cases were compared to the results from the algorithm to determine the positive predictive value (PPV) of the algorithm for prevalent ILD, and a separate algorithm for incident ILD. Incident ILD cases were considered to be correctly classified if the ILD onset date per the medical record review was within +/− 6 months of the ILD case date as identified by the claims-based algorithm.

### ILD algorithm definition

Drawing from the literature, the study team created a list of ICD-9-CM and ICD-10-CM codes that could potentially indicate the presence of an ILD diagnosis (Supplemental Table 1). The team further divided these codes into Specific (bolded) and Sensitive (non-bolded) conditions. Searching the Medicare data, we required ICD-9-CM or ICD-10-CM diagnosis code for ILD from an inpatient hospitalization claim in any position (primary or non-primary position), OR one or more outpatient diagnosis codes for ILD from a pulmonologist, rheumatologist, or internist, plus an outpatient chest computed tomography (CT), or outpatient lung biopsy, or any hospitalization in the preceding 90 days. Similar algorithms have been used previously in other claims-based studies [8] and recently validated in patients with RA in the VA health system [14]. The rationale behind allowing for a recent hospitalization to act as a surrogate for an outpatient CT scan is that many diagnostic tests (like this one) performed on hospitalized patients are not separately recorded in the data.

To identify incident ILD, we applied further exclusion criteria to the aforementioned algorithm. Given the expectation that a period free of any ILD or other pulmonary diagnoses for approximately 2 years would be required to appropriately classify incident ILD (Fig. 2a, b), we used all available data prior to the index date with a minimum requirement of at least 12 months. Secondly, we evaluated all data 12 months after the index date such that we had a minimum of a two-year ascertainment period to identify and exclude prevalent ILD. During these time periods, we required the patient to have no ILD diagnosis codes, indicators of prevalent ILD (e.g., lung biopsy), or diagnosis codes for sarcoidosis. However, because ILD might require several months to evaluate and ultimately diagnose, we allowed evidence for ILD to accrue up to 6 months prior to the confirmed ILD event date. At least one ILD case qualifying event date must have occurred after the RA Cohort Index date, consistent with the goal of identifying ILD in a cohort of patients already classified as having RA according to validated approaches [17].

### Statistical analysis

Descriptive statistics were generated for the cohort, stratified by ILD case status (confirmed, not confirmed, or insufficient information to determine). Imbalances in characteristics at *p* < 0.05 and with standardized mean differences (SMDs) > 0.10 were considered potentially clinically meaningful. We calculated positive predictive values (PPV) of the ILD algorithm with 95% confidence intervals estimated using a binomial approximation. The PPV was calculated for the ILD algorithm for any case of ILD (prevalent or incident), compared to ILD classification by medical record review. We also assessed the PPV of the incident ILD algorithm criteria in finding incident ILD, both with conditioning on having prevalent ILD and without conditioning as well. Finally, we examined seropositive RA associated with ILD, using an ICD-10-CM diagnosis code M05 (rheumatoid arthritis with rheumatoid factor), which has previously been shown to reasonably proxy for positive rheumatoid factor and/or anti-CCP antibody [18].

## Results

### Cohort identification and characteristics

**Fig. 1:**
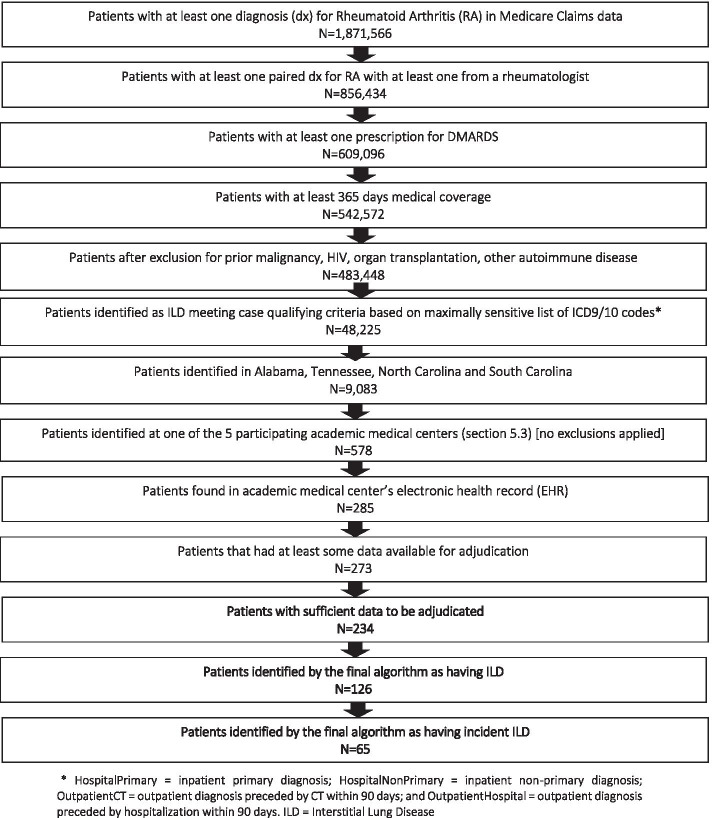
Flow diagram for selecting ILD cases according to a claims-based algorithm

After applying inclusion and exclusion criteria to the Medicare data and restricting to the five participating medical centers, we identified 578 RA patients receiving any care at the participating locations (51 at VUMC, 51 at MUSC, 243 at UNC, 170 at Duke, and 63 at UAB). Of these patients, 273 were linkable and had at least some data available for the evaluation of ILD, and 234 had sufficient data to allow for adjudication of ILD. The 39 cases that did not have sufficient information for clinical adjudication of ILD status were excluded from further analysis, leaving 234 RA patients with sufficient data to be evaluated for ILD (Fig. 1). The patient population had a mean age of 66 years, was predominately female (67%) and white (73%). Based on the weighted Charlson index [19], 62% had a score of 1-2. Diabetes and various manifestations of cardiovascular disease were the most common comorbidities. Approximately 19% of patients were classified as smokers based on diagnosis codes or prescribed smoking cessation therapies occurring in the claims data. In addition, 36% of patients had prior use of methotrexate, 40% of biologic DMARDS, and 64% of glucocorticoids. There were no significant and meaningful differences in the characteristics of patients who had their ILD confirmed, unconfirmed, or were unable to be classified (Table 1).

**Table 1:** Characteristics of confirmed vs. unconfirmed ILD cases following clinical adjudication

	Yes, confirmed ILD	No, not confirmed ILD	Insufficient information to determine	***P*** value	SMD *(comparing all three groups)*
***N***	108	126	39		
Age (mean (sd))	66 (9.7)	67 (9.6)	67 (10.2)	0.80	0.06
Male sex (%)	36 (33.3)	32 (25.4)	7 (17.9)	0.14	0.24
Race (%)				0.52	0.34
White	78 (72.9)	92 (73.6)	32 (82.1)	-	-
Black	22 (20.6)	21 (16.8)	7 (17.9)	-	-
Unknown	5 (4.7)	10 (8.0)	0 (0.0)	-	-
Other	2 (1.9)	2 (1.6)	0 (0.0)	-	-
Weighted Charlson index^a^ (mean (sd))	2 (1.5)	2 (1.5)	2 (1.9)	0.95	0.03
Comorbidities					
Cerebrovascular disease (%)	8 (7.4)	11 (8.7)	2 (5.1)	0.75	0.10
Congestive heart failure (%)	15 (13.9)	21 (16.7)	7 (17.9)	0.78	0.07
Diabetes without complications (%)	26 (24.1)	18 (14.3)	11 (28.2)	0.07	0.23
Diabetes with complications (%)	8 (7.4)	6 (4.8)	2 (5.1)	0.68	0.07
Myocardial infarction (%)	9 (8.3)	6 (4.8)	2 (5.1)	0.51	0.10
Peripheral vascular disease (%)	11 (10.2)	14 (11.1)	3 (7.7)	0.83	0.08
Renal disease (%)	9 (8.3)	14 (11.1)	5 (12.8)	0.67	0.10
Any tobacco use (%)	21 (19.4)	30 (23.8)	11 (28.2)	0.49	0.14
RA Medications, any prior use (%)					
Methotrexate (%)	39 (36.1)	56 (44.4)	20 (51.3)	0.20	0.21
Biologic DMARDS (%)	43 (39.8)	49 (38.9)	10 (25.6)	0.26	0.20
Glucocorticoids, e.g., prednisone (%)	69 (63.9)	70 (55.6)	23 (59.0)	0.43	0.11

Note: All variables in the Charlson and each of the specific comorbidities were ascertained using the 12 months prior to the index date. All other variables including tobacco use and RA medications were classified based on all prior historical data available

*ILD* interstitial lung disease, *SMD* standardized mean difference

^a^Excluding RA in the Charlson weighting

Using Medicare claims data, the characteristics of suspected ILD cases that could vs. could not be linked to the EHR with sufficient clinical data are shown in Supplemental Table 2. The differences between cases with and without EHR data enabling clinical adjudication were minimal (SMDs < 0.10), with the exception that cases with available EHR data were slightly more likely to be tobacco users, and slightly less likely to use methotrexate and biologics.

### ILD based on Adjudication

Overall, 108 (46.2%) of preliminarily suspected cases (based on our highly sensitive case-finding approach) were classified as ILD based on adjudication (Supplemental Table 3). The most common diagnosis codes identified in this initial search were ICD-9-CM code 515 (post inflammatory pulmonary fibrosis) with 83 out of 234 cases (35.5%) and ICD-9-CM 518.89 (other disorders of the lung) with 47 out of 234 (20.1%). When comparing the accuracy of the codes for case finding, 515 had the best PPV at 78% (65/83 cases); 714.81 (rheumatoid lung) had the next best at 67% (12/18 cases). Conversely, 494.0 (bronchiectasis), 518.89 (other disorders of lung), and 793.19 (other nonspecific abnormal findings of lung field) all performed poorly (PPVs of 5–33%)

### Performance of algorithm to classify both prevalent and incident ILD

As shown in Table 2, the final ILD algorithm for any incident or prevalent ILD had a PPV of 77% (95% CI 69–84%). This estimate was based on the ILD algorithm having identified 126 cases of ILD, and the gold standard adjudication confirming 97 of those cases. The sensitivity of the algorithm compared to the broader case-finding approach (based on single diagnosis codes for ILD) was 90% (95% CI 83–95%).

**Table 2:** Positive predictive value of final ILD algorithm for incident or prevalent ILD compared to gold standard of clinical adjudication (*N* = 234 suspected cases with sufficient data)

	ILD based on adjudication (gold standard)			
Algorithm^a^ to find all cases of ILD (prevalent or incident)	Yes	No	Total	Positive predictive value, 95% confidence interval
Yes, All cases	97	29	126	77%, 69–84%
HospitalPrimary	8	3	11	73%, 39–94%
HospitalNonPrimary	22	14	36	61%, 44–77%
Outpatient	67	12	79	85%, 77–91%^b^
OutpatientCT	61	10	71	86%, 76–93%
OutpatientHospital	6	2	8	75%, 35–97%
No	11	97	108	
Total	108	126	234	

*ILD* interstitial lung disease

Note: Baseline exclusions for the RA cohort excluded those with malignancy and other autoimmune diseases

Estimated sensitivity = 97/108 = 90% (95% CI 83–95%) [among those who initially fulfilled screening algorithm]

^a^All ICD-9/10-CM codes for ILD listed in Table 3 were included

^b^If 2 outpatient ILD diagnosis codes were required (*n*=59 cases in this sample), the PPV of OutpatientCT or OutpatientHospital cases was 86% (95% CI 76–93%)

### Performance of ILD algorithm by case qualifying definition

A majority of ILD cases (79/126, 63%) were diagnosed in the outpatient setting by 68 unique providers. OutpatientCT cases (i.e. outpatient diagnosis preceded by CT) captured the largest proportion of validated ILD diagnoses (61/97, 63%). The PPVs according to case qualifying status were highest for Outpatient cases (85%, 95% CI 77–91%) and lowest for HospitalNonPrimary cases (PPV=61%, 95% CI 44–77%). For Outpatient cases, if a second diagnosis code was required within 365 days (*n* = 59, 75% of all Outpatient cases), the PPV increased only slightly (86%, 95% CI 76–93%)see footnote in Table 2. The Outpatient diagnosed cases always had the highest PPV, particularly those that were preceded by a CT scan, yielding a PPV of 86% (including the non-confirmed ILD cases). Most of the outpatient diagnosed cases were diagnosed by a pulmonologist (*n*=56), with fewer diagnosed by a rheumatologist (*n*=12) or an internist (*n*=11). Additional criteria such as exclusions for prior use of home-based oxygen (as a proxy for prevalent ILD) did not affect the disposition of any ILD case (data not shown). The diagnoses codes that were used in our final ILD algorithm to find incident or prevalent ILD are shown in Table 3. For cases where ICD-10-CM diagnosis codes were available (*n*=118 cases), 66% of confirmed cases were seropositive according to M05 diagnosis code(s), a higher proportion than those that were not confirmed (46%, *p* = 0.03).

**Table 3:** Frequency of specific ICD-9/10-CM ILD Codes using the final ILD case-finding algorithm

Table of diagnosis by ILD code					
Diagnosis code and location of case qualifying^a^ ILD		Adjudicated ILD			
		Yes, incident	Yes, prevalent	Not ILD	Total^**b**^
515, Post inflammatory pulmonary fibrosis	HospitalPrimary	4	1	2	7
	HospitalNonPrimary	12	7	13	32
	OutpatientCT	33	9	8	50
	OutpatientHospital	4	2	1	7
516.31, Idiopathic pulmonary fibrosis	HospitalNonPrimary	1	0	0	1
	OutpatientCT	2	0	0	2
516.34, Respiratory bronchiolitis interstitial lung disease	OutpatientCT	0	1	0	1
	OutpatientHospital	0	0	1	1
516.8, Other specified alveolar and parietoalveolar pneumonopathies	HospitalPrimary	1	0	0	1
	HospitalNonPrimary	0	1	0	1
	OutpatientCT	1	2	0	3
714.81, Rheumatoid lung disease	HospitalPrimary	1	0	1	2
	OutpatientCT	6	3	2	11
J84.10, Pulmonary fibrosis unspecified	HospitalNonPrimary	1	0	0	1
J84.112, Idiopathic pulmonary fibrosis	OutpatientCT	1	0	0	1
J84.9, Interstitial pulmonary disease, unspecified	HospitalNonPrimary	0	0	1	1
	OutpatientCT	2	1	0	3
M05.10, Rheumatoid lung disease	HospitalPrimary	0	1	0	1
Total		69	28	29	126

*ILD* interstitial lung disease

^a^ HospitalPrimary = inpatient primary diagnosis; HospitalNonPrimary = inpatient non-primary diagnosis; OutpatientCT = outpatient diagnosis preceded by CT within 90 days; and OutpatientHospital = outpatient diagnosis preceded by hospitalization within 90 days

^b^The count of each diagnosis code row in Table 3 may exceed the corresponding row counts in Supplemental Table 3, since Supplemental Table 3 shows the counts for all codes used to initially screen for ILD based on patients’ first ILD diagnosis appearing in the data. Table 3 reflects only ILD diagnosis codes included in the Final ILD algorithm, eliminating those that performed poorly. Patients commonly had multiple ILD diagnosis codes over time; those initially found in the data may be been re-categorized by the final algorithm. For example, if a patient’s first ILD diagnosis code was 491.9, then they would have been included in that count for Supplemental Table 3. However, in Table 3, this code was eliminated, so those patients would have been represented in Table 3 according to whatever their ILD diagnosis code was that appeared in the final ILD algorithm

Furthermore, ICD-9-CM code 515 remained the most commonly occurring single diagnosis code overall to capture the largest number of true cases (72 confirmed ILD cases out of 96 possible) and by case qualification criteria (42 out of 50 OutpatientCT cases adjudicated as ILD) (Table 3). A summary of the most commonly appearing ICD-10-CM codes assigned by pulmonologists and rheumatologists among confirmed cases initially ascertained using ICD-9-CM coding were in the J84 code group (Supplemental Table 4). A minority were identified as M05.1 (rheumatoid lung disease with rheumatoid arthritis), J18.9 (pneumonia or pneumonitis, unspecified organism), and R91.8 (other nonspecific abnormal findings of lung field).

**Fig. 2:**
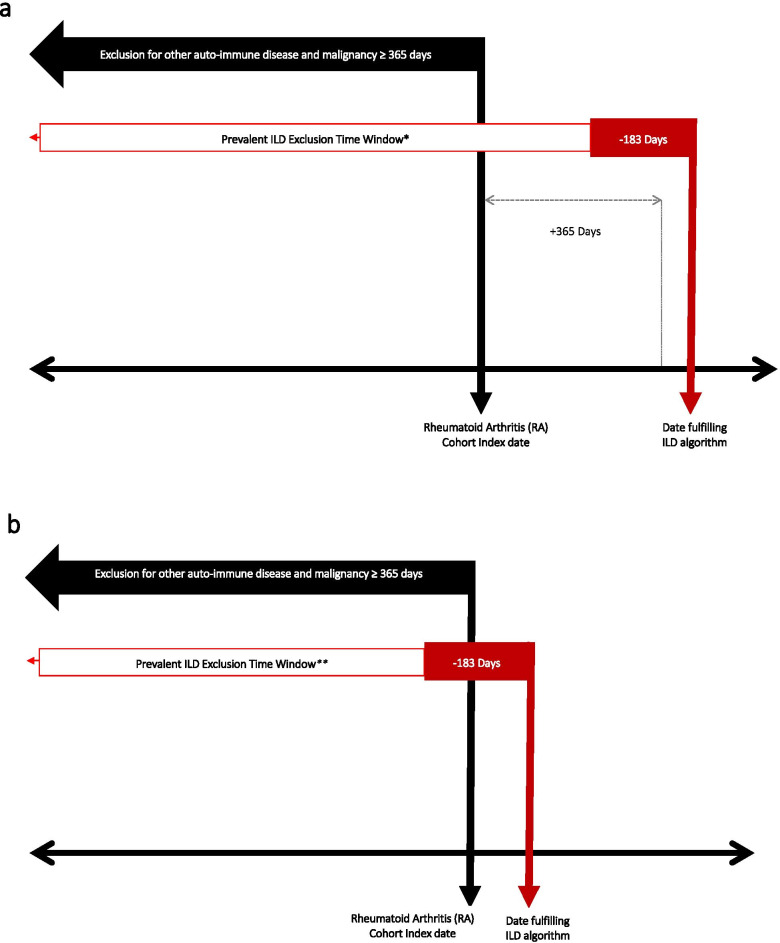
**a** Study time windows to exclude prevalent ILD and other pulmonary conditions to find incident ILD, event date occurring >365 days after the RA Cohort Index Date. * For the incident ILD algorithm, in addition to the exclusions for autoimmune disease and malignancy shown above, additional exclusions were made for Specific ILD Codes (exhaustive list in Supplemental Table 1) plus ICD-9-CM 135 (Sarcoidosis), and ICD-10-CM D86.9 (Sarcoidosis). The red box indicates a 6-month interval of time during which ILD-related diagnoses can accrue, even if the patient has not yet met ILD case qualifying criteria. ILD, interstitial lung disease. **b** Study time windows to exclude prevalent ILD and other pulmonary conditions to find incident ILD identified within 365 days of eligibility for the RA Cohort. *Incident ILD cases could occur even if the date fulfilling the ILD algorithm was within the +365 day period following the RA cohort index date. The Prevalent ILD Exclusion Time Window is applied prior to the RA Cohort Index date, and this step will exclude prevalent ILD cases. The red box indicates a 6-month interval of time during which ILD-related diagnoses can accrue. ** For incident ILD algorithm, in addition to the exclusions for autoimmune disease and malignancy, additional exclusions were made for Specific ILD Codes (exhaustive list in Supplemental Table 1) plus ICD-9-CM 135 (Sarcoidosis), and ICD-10-CM D86.9 (Sarcoidosis). ILD, interstitial lung disease

The algorithm for incident ILD was the same as the final ILD algorithm, with additional exclusions applied that required a more extended “clean” period of approximately 2 or more years. Figure 2a describes the circumstance where an RA patient develops incident ILD one or more years after eligibility for the RA cohort. Figure 2b describes the circumstance where someone is identified as having both RA and also develops incident ILD at approximately the same time, with the prevalent ILD exclusion applied prior to qualifying for the RA cohort. The performance of the incident ILD algorithm is shown in Table 4, with details of the algorithm shown in the footnote. Among patients who were confirmed as having ILD, the PPV of the additional requirements found in the incident ILD algorithm for classifying incident vs. prevalent disease was 96% (95% CI 85–100%). Incorporating all ILD cases, including those not confirmed to be prevalent or incident ILD by clinical adjudication, the overall PPV was 69% (95% CI 57–80%). If hospitalized cases with ILD diagnoses appearing in the non-primary position were excluded (i.e., HospitalNonPrimary cases), the PPV increased to 82% (95% CI 68–92%).

**Table 4:** Positive predictive value of final algorithm for incident ILD according to varying case qualification definitions

		ILD according to adjudicated gold standard						
	Case qualifying ILD definition^b^	Incident	Prevalent	Any ILD (incident + prevalent)	PPV for incident ILD, conditional on confirmed case of ILD	Not ILD	Total (any ILD + not ILD)	PPV for incident ILD, all cases
Final incident ILD algorithm^a^	Yes, HospitalPrimary	6	1	7	86% (42%, 100%)	1	8	75% (35%, 97%)
	Yes, HospitalNonPrimary	8	1	9	89% (52%, 100%)	11	20	40% (19%, 64%)
	Yes, OutpatientCT	28	0	28	100% (88%, 100%)	5	33	85% (68%, 95%)
	Yes, OutpatientHospital	3	0	3	100% (29%, 100%)	1	4	75% (19%, 99%)
	Yes, All definitions	45	2	47	96% (85%, 100%)	18	65	69% (57%, 80%)
	Total without HospitalNonPrimary ILD Cases	37	1	38	97% (86%, 100%)	7	45	82% (68%, 92%)

*ILD* interstitial lung disease

^a^The algorithm excluded cases with ICD-9-CM 135 (Sarcoidosis), ICD-10-CM D869 (Sarcoidosis), and ILD Definition Codes (exhaustive list in section 5.1) through minimum of (Index Date +365 days, Outcome Date −183 days)

^b^HospitalPrimary = inpatient primary diagnosis; HospitalNonPrimary = inpatient non-primary diagnosis; OutpatientCT = outpatient diagnosis preceded by CT; and OutpatientHospital = outpatient diagnosis preceded by hospitalization

## Discussion

Using national Medicare data, we developed and validated a claims-based algorithm to identify patients with incident RA-ILD, and confirmed the generalizability of prior, similar approaches to find prevalent ILD in more restricted data systems (e.g., the US Veterans Health Administration). Based on linkage with medical records for 234 RA patients, the best performing algorithm to ascertain incident or prevalent ILD had a PPV of 77% (95% CI 69–84%). When refining the algorithm to require ≥ 2 ILD diagnosis codes in an outpatient setting, the PPV increased to 86%. Further refinement to exclude prevalent RA-ILD and conditioning on confirmed ILD cases only increased the PPV for incident RA-ILD to 96% (95% CI 85–100%). Overall, the algorithm for incident ILD had a PPV of 69% (95% CI 57–80%).

Our validation of prevalent RA-ILD algorithms yielded similar PPVs to what has been previously reported in more limited healthcare systems. In one prior study, validation was done for prevalent RA-ILD diagnosis codes in a Kaiser Permanente Northern California study using CT and chest x-ray reports for validation without additional medical record review [13]. This study found a PPV of 63% for ≥ 2 ILD diagnosis codes. In another study, several prevalent ILD coding algorithms were validated by medical record review within a Veterans Affairs RA cohort and yielded PPVs ranging from 66 to 86% [14]. Our study yielded a PPV of 77% for prevalent ILD which is consistent with these prior validation studies. This work extends validation to Medicare enrollees which includes a diverse national US population. Perhaps of even higher importance, algorithms for incident RA-ILD have previously been used in administrative datasets [1, 8, 9], but this is the first study to validate an administrative algorithm for classifying incident RA-ILD. Our algorithm was highly accurate at differentiating incident vs. prevalent RA-ILD with a PPV of 96% among confirmed cases. Deployed across the entire claim’s dataset, this algorithm retained a PPV of 69%. The difference between these two PPVs indicates that our approach to classify ILD as new-onset was highly accurate, and the majority of the misclassification was due to whether the patient had confirmed ILD or not. We would also note that the prior VA study performed ILD identification in a cohort that was known to have RA. Our approach went an additional step in that it combined administrative algorithms for both RA and ILD, and compared these to a clinical gold standard for ILD, demonstrating similarly high accuracy to classify prevalent and incident ILD. These validated administrative data definitions for RA-ILD will assist researchers in both measuring RA-ILD incidence and assessing the features and patterns of care of new-onset disease in a systematic fashion.

### Limitations

As with any retrospective observational study, there are limitations. The most common limitation in this study was missing or inaccurate data recorded in claims data (e.g. incorrect date of birth making positive identification of some patients uncertain) or in the EHR. To address this issue, we matched patients using other factors including dates of service and sex, but for this reason, some patients could not be matched using deterministic methods. The analyses sometimes were limited by a lack of the desired data for ILD confirmation or classification of incident vs. prevalent ILD in circumstances where one of the patient’s providers was not associated with the medical system. These cases were included in the analyses, but with an annotation that the adjudicators could not ascertain these cases as incident or prevalent given uncertainties regarding timing. Furthermore, the focus of this study was on clinically recognized and diagnosed ILD. Patients may have had a subclinical disease that was asymptomatic and, therefore, not evaluated and detected. Similarly, “incident” ILD was operationalized as newly diagnosed but this concept does not necessarily refer to the time of actual onset of subclinical disease, which would essentially be impossible in the absence of a prospective, population-based screening strategy. Finally, we have focused our evaluation on the PPV of incident and prevalent ILD, but have not reported negative predictive values (NPV). To compute a NPV, one needs an orthogonal method to find ILD cases that are independent of the diagnosis-based approach that we used. This would typically require a bespoke ILD registry (not available at most of the medical centers included in this study), or manually reviewing all data for a randomly selected number of RA patients for whom there was no evidence of ILD or pulmonary disease, a resource-intensive endeavor that was expected to have low yield.

Our study used claims data from Medicare and thereby identified cases in predominantly older people and/or those with severe rheumatoid arthritis (based on qualifying for Medicare for reasons of disability, presumably due to RA). Thus, these results may not generalize to younger patients or those with less antecedent data. Because of our study period, the project focused primarily on ICD-9-CM codes (only seven patients from September to December of 2015 included in the study that presented with an ICD-10-CM code, but no previous ICD-9-CM diagnosis). We provided a descriptive summary of ICD-10-CM codes for confirmed cases (Supplemental Table 4) to aid in mapping the ICD-9-CM diagnosis codes to their ICD-10-CM equivalents. The set of pulmonary conditions that might be initially mistaken for ILD (e.g., sarcoidosis) that were used as exclusions for incident ILD during the baseline period may need to be expanded to include a few additional uncommon conditions (e.g., asbestosis, pneumoconiosis, ICD-9-CM 505), depending on the prevalence of these in the population under study. We also recognize that it is possible that ILD was diagnosed prior to the RA Cohort Index date. While our interest was to identify prevalent and incident ILD in a cohort of patients already classified as having RA, additional variants on this algorithm might (for example) allow ILD to be identified shortly prior to meeting RA cohort inclusion criteria. This variant likely would be most useful when coupled with an inception cohort of RA patients, a feature not particularly relevant for our study cohort with mean age 67 years for whom most patients were expected to have prevalent RA. Finally, although having five participating centers represents a strength of this project, relative to the overall number of academic medical centers in the USA, this number is relatively small and coding practices may differ from institution to institution.

## Conclusion

In conclusion, leveraging a nationally diverse US population in Medicare, we found that ILD case finding among RA patients using administrative claims data is feasible with a high degree of accuracy (PPV=77%). In addition to establishing the external validity of other approaches [13, 14], we have, for the first time, extended such algorithms to differentiate incident vs. prevalent RA-ILD in a highly accurate manner (PPV=96%). Equipped with these algorithms, researchers can harness claims datasets for epidemiologic and outcomes research in RA-ILD.

## Supplementary Information

**Additional file 1: Table S1**: Specific (Bold) and Sensitive (Not Bold) Diagnosis codes suggestive of Interstitial Lung Disease.

**Additional file 2: Table S2**: Characteristics of All Cases (N=578).

**Additional file 3: Table S3**: Positive Predictive Value of Individual ICD-9 /10-CM Codes Initially Screened for ILD (n=234) Prior to Application of Final ILD Algorithm.

**Additional file 4: Table S4**: ICD-10-CM Codes Appearing on Outpatient Visit Claims Among Confirmed ILD Cases Initially Identified Based on ICD-9-CM Codes.

## Data Availability

The datasets used and/or analyzed during the current study are available from the corresponding author upon reasonable request.
