# Supplementary material for: Validation of an algorithm to identify incident interstitial lung disease in patients with rheumatoid arthritis
Source: Arthritis Res Ther. 2022 Jan 3;24:2. doi: 10.1186/s13075-021-02655-z (PMC8722182; doi:10.1186/s13075-021-02655-z)
Supplement: Supplementary file 1 — Additional file 1: Table S1: Specific (Bold) and Sensitive (Not Bold) Diagnosis codes suggestive of Interstitial Lung Disease. [file 13075_2021_2655_MOESM1_ESM.docx]

## Supplemental Table 1: Specific (Bold) and Sensitive (Not Bold) Diagnosis codes suggestive of Interstitial Lung Disease

| **ICD-9-CM Codes (through 9/30/2015)** |
| --- |
| - 491.8 (Other chronic bronchitis) |
| - 491.9 (Unspecified chronic bronchitis) |
| - 494.0 (Bronchiectasis) |
| - **495.9 (Unspecified allergic alveolitis and pneumonitis)** |
| - **515 (Post inflammatory pulmonary fibrosis)** |
| - **516.30 (Idiopathic interstitial pneumonia, NOS)** |
| - 516.31 (Idiopathic pulmonary fibrosis) |
| - 516.32 (Idiopathic nonspecific interstitial pneumonitis) |
| - 516.33 (Acute interstitial pneumonitis) |
| - **516.34 (Respiratory bronchiolitis interstitial lung disease)** |
| - 516.35 (Idiopathic lymphocytic interstitial pneumonitis |
| - 516.36 (Cryptogenic organizing pneumonia) |
| - **516.37 (Desquamative interstitial pneumonia)** |
| - **516.8 (Other specificed alveolar and parietoalveolar pneumonopathies)** |
| - **516.9 (Unspecificed alveolar and parietoalveolar pneumonopathy)** |
| - **517.8 (Lung involvement diseases classified elsewhere)** |
| - 518.89 (Other diseases of lung, not elsewhere classified) |
| - **714.81 (Rheumatoid lung disease)** |
| - 793.19 (Other nonspecific abnormal finding of lung field) |
| **ICD-10-CM Codes (10/1/2015 and after)** |
| - J18.9 (Pneumonitis, drug induced pneumonitis) |
| - J42 (Unspecified chronic bronchitis) |
| - J47.9 (Bronchiectasis) |
| - J67.9 (Hypersensitivity pneumonitis due to unspecified organic dust) |
| - J84.09 (Other alveolar and parieto-alveolar conditions) |
| - **J84.10 (Pulmonary fibrosis unspecified)** |
| - **J84.115 (Respiratory bronchiolitis interstitial lung disease)** |
| - **J84.117 (Desquamative interstitial pneumonia)** |
| - **J84.89 (Other specified interstitial pulmonary disease)** |
| - **J84.9 (Interstitial pulmonary disease, unspecified)** |
| - **M05.10-17 (Rheumatoid lung disease with rheumatoid arthritis)** |
| - **M05.19 (Rheumatoid lung disease with rheumatoid arthritis of multiple sites)** |
| - R91.8 (Other nonspecific abnormal finding of lung field) |

Specific definition codes are bolded. Sensitive definition includes all codes, bold and non-bold.
