# Supplementary material for: Validation of an algorithm to identify incident interstitial lung disease in patients with rheumatoid arthritis
Source: Arthritis Res Ther. 2022 Jan 3;24:2. doi: 10.1186/s13075-021-02655-z (PMC8722182; doi:10.1186/s13075-021-02655-z)
Supplement: Supplementary file 2 — Additional file 2: Table S2: Characteristics of All Cases (N=578). [file 13075_2021_2655_MOESM2_ESM.docx]

Supplemental Table 2: Characteristics of All Cases (N=578)

|  | **Cases not linked to EHR and/or with no data** | **Cases with available data** | **P value** | **SMD** *(comparing both groups)* |
| --- | --- | --- | --- | --- |
| **N** | **305** | **273** |  |  |
| **Age (mean (sd))** | 66.91 (10.36) | 66.38 (9.70) | 0.52 | 0.05 |
| **Male Sex (%)** | 80 (26.2) | 75 (27.5) | 0.81 | 0.03 |
| **Race (%)** |  |  | 0.21 | 0.25 |
| White | 231 ( 75.7) | 202 ( 74.5) | - | - |
| Black | 55 ( 18.0) | 50 ( 18.5) | -  Incident ILD correctly classified by algorithm if case date is 6+/- months from onset date in medical record | - |
| Unknown | 8 ( 2.6) | 17 ( 5.5) | -  Incident ILD correctly classified by algorithm if case date is 6+/- months from onset date in medical record | - |
| Other | 11 (3.7) | 4 (1.5) | -  Incident ILD correctly classified by algorithm if case date is 6+/- months from onset date in medical record | - |
| **Weighted Charlson index* (mean (sd))** | 2.50 (1.44) | 2.45 (1.58) | 0.75 | 0.03 |
| **Comorbidities** |  |  |  |  |
| Cerebrovascular disease (%) | 22 (7.2) | 21 ( 7.7) | 0.95 | 0.02 |
| Congestive heart failure (%) | 43 (14.1) | 43 ( 15.8) | 0.66 | 0.05 |
| Chronic pulmonary disease (%) | 144 (47.2) | 132 ( 48.4) | 0.85 | 0.02 |
| Diabetes without complications (%) | 62 (20.3) | 55 ( 20.1) | 1.00 | 0.00 |
| Diabetes with complications (%) | 25 (8.2) | 16 ( 5.9) | 0.35 | 0.09 |
| Myocardial infarction (%) | 16 (5.2) | 17 ( 6.2) | 0.74 | 0.04 |
| Peripheral vascular disease (%) | 32 (10.5) | 28 ( 10.3) | 1.00 | 0.01 |
| Renal disease (%) | 29 (9.5) | 28 ( 10.3) | 0.87 | 0.03 |
|  |  |  |  |  |
| **Any Tobacco Use (%)** | 54 ( 17.7) | 62 ( 22.7) | 0.16 | 0.12 |
| **RA Medications, any prior use (%)** |  |  |  |  |
| Methotrexate (%) | 151 (49.5) | 115 ( 42.1) | 0.09 | 0.15 |
| Biologic DMARDS (%) | 137 (44.9) | 102 ( 37.4) | 0.08 | 0.15 |
| Glucocorticoids e.g. prednisone (%) | 181 (59.3) | 162 ( 59.3) | 1.00 | 0.00 |

*excluding RA in the Charlson weighting [19]

ILD = Interstitial Lung Disease; SMD =Standardized Mean Difference
