# Supplementary material for: Validation of an algorithm to identify incident interstitial lung disease in patients with rheumatoid arthritis
Source: Arthritis Res Ther. 2022 Jan 3;24:2. doi: 10.1186/s13075-021-02655-z (PMC8722182; doi:10.1186/s13075-021-02655-z)
Supplement: Supplementary file 3 — Additional file 3: Table S3: Positive Predictive Value of Individual ICD-9 /10-CM Codes Initially Screened for ILD (n=234) Prior to Application of Final ILD Algorithm. [file 13075_2021_2655_MOESM3_ESM.docx]

**Supplemental Table 3: Positive Predictive Value of Individual ICD-9 /10-CM Codes Initially Screened for ILD (n=234) Prior to Application of Final ILD Algorithm**

| **Diagnosis Code and Case Qualifying (CQ) Status*** | **Adjudicated ILD (may be incident or prevalent)** | | |
| --- | --- | --- | --- |
|  | **Confirmed, n (%)** | **Not Confirmed, n (%)** | **Total, n** |
| **515, Post inflammatory pulmonary fibrosis** |  |  |  |
| HospitalPrimary (n=6), HospitalNonPrimary (n=26), OutpatientCT (n=45), OutpatientHospital (n=6) | 65 (78%) | 18 (22%) | 83 |
| **714.81, Rheumatoid lung disease** |  |  |  |
| HospitalPrimary (n=2); HospitalNonPrimary (n=4); OutpatientCT (n=10); OutpatientHospital (n=2) | 12 (67%) | 6 (33%) | 18 |
| **494.0, Bronchiectasis** |  |  |  |
| HospitalNonPrimary (n=13); OutpatientCT (n=11); OutpatientHospital (n=2) | 9 (35%) | 17 (65%) | 26 |
| **518.89, Other diseases of lung, not elsewhere classified** |  |  |  |
| HospitalPrimary (n=2); HospitalNonPrimary (n=12); OutpatientCT (n=30); OutpatientHospital (n=3) | 8 (17%) | 39 (83%) | 47 |
| **516.8, Other specificed alveolar and parietoalveolar pneumonopathies** |  |  |  |
| HospitalPrimary (n=1); OutpatientCT (n=3) | 4 (100%) | 0 (0%) | 4 |
| **J84.9, Interstitial pulmonary disease, unspecified** |  |  |  |
| OutpatientCT (n=3) | 3 (100%) | 0 (0%) | 3 |
| **516.31, Idiopathic pulmonary fibrosis** |  |  |  |
| HospitalNonPrimary (n=1); OutpatientCT (n=1) | 2 (100%) | 0 (0%) | 2 |
| **516.34, Respiratory bronchiolitis interstitial lung disease** |  |  |  |
| OutpatientCT (n=1) | 1 (100%) | 0 (0%) | 1 |
| **491.8, Other chronic bronchitis** |  |  |  |
| OutpatientCT (n=1) | 1 (100%) | 0 (0%) | 1 |
| **793.19, Other nonspecific abnormal finding of lung field** |  |  |  |
| HospitalNonPrimary (n=7): OutpatientCT (n=11); OutpatientHospital (n=4) | 1 (5%) | 21 (96%) | 22 |
| **J47.9, Bronchiectasis** |  |  |  |
| OutpatientCT (n=1) | 1 (100%) | 0 (0%) | 1 |
| **J84.10, Pulmonary fibrosis unspecified** |  |  |  |
| HospitalNonPrimary (n=1) | 1 (100%) | 0 (0%) | 1 |
| **491.9, Unspecified chronic bronchitis** |  |  |  |
| HospitalNonPrimary (n=3); OutpatientCT (n=2) | 0 (0%) | 5 (100%) | 5 |
| **516.30, Idiopathic interstitial pneumonia, NOS** |  |  |  |
| OutpatientCT (n=1) | 0 (0%) | 1 (100%) | 1 |
| **517.8, Lung involvement diseases classified elsewhere** |  |  |  |
| HospitalNonPrimary (n=4); OutpatientCT (n=1); OutpatientHospital (n=1) | 0 (0%) | 6 (100%) | 6 |
| **J18.9, Pneumonitis, drug induced pneumonitis** |  |  |  |
| HospitalPrimary (n=2); HospitalNonPrimary (n=8); OutpatientHospital (n=1) | 0 (0%) | 11 (100%) | 11 |
| **R91.8, Other nonspecific abnormal finding of lung field** |  |  |  |
| OutpatientCT (n=2) | 0 (0%) | 2 (100%) | 2 |
| **Total** | **108 (46.2%)** | **126 (53.8%)** | **234** |

Data shown as Frequency (Row Percent i.e. Positive Predictive Value for confirmed column); ILD = Interstitial Lung Disease
* HospitalPrimary = inpatient primary diagnosis; HospitalNonPrimary = inpatient non-primary diagnosis; OutpatientCT = outpatient diagnosis preceded by CT within 90 days; and OutpatientHospital = outpatient diagnosis preceded by hospitalization within 90 days
