# Supplementary material for: Validation of an algorithm to identify incident interstitial lung disease in patients with rheumatoid arthritis
Source: Arthritis Res Ther. 2022 Jan 3;24:2. doi: 10.1186/s13075-021-02655-z (PMC8722182; doi:10.1186/s13075-021-02655-z)
Supplement: Supplementary file 4 — Additional file 4: Table S4: ICD-10-CM Codes Appearing on Outpatient Visit Claims Among Confirmed ILD Cases Initially Identified Based on ICD-9-CM Codes. [file 13075_2021_2655_MOESM4_ESM.docx]

Supplemental Table 4: ICD-10-CM Codes Appearing on Outpatient Visit Claims Among Confirmed ILD Cases Initially Identified Based on ICD-9-CM Codes

| **Table of Diagnosis by ILD Code** | | | | | | | | | | | |
| --- | --- | --- | --- | --- | --- | --- | --- | --- | --- | --- | --- |
| **Diagnosis Code and Location of Case Qualifying* ILD** | **J18.9 Pneumonitis, drug induced pneumonitis** | **J84.09 Other alveolar and parieto-alveolar conditions** | **J84.10 Pulmonary fibrosis unspecified** | **J84.115 Respiratory bronchiolitis interstitial lung disease** | **JJ84.89 Other specified interstitial pulmonary disease** | **J84.9 Interstitial pulmonary disease, unspecified** | **M05.10 Rheumatoid lung disease with rheumatoid arthritis** | **M05.19 Rheumatoid lung disease with rheumatoid arthritis of multiple sites** | **R91.8 Other nonspecific abnormal finding of lung field** | **No ICD10 code**** | **Total** |
| **515, Post inflammatory pulmonary fibrosis** | 7 | 0 | 15 | 1 | 1 | 15 | 7 | 4 | 4 | 42 | 96 |
| **516.31, Idiopathic pulmonary fibrosis** | 0 | 0 | 0 | 0 | 0 | 1 | 0 | 1 | 0 | 1 | 3 |
| **516.34, Respiratory bronchiolitis interstitial lung disease** | 1 | 0 | 0 | 0 | 0 | 0 | 0 | 0 | 0 | 1 | 2 |
| **516.8, Other specificed alveolar and parietoalveolar pneumonopathies** | 0 | 1 | 2 | 0 | 0 | 0 | 0 | 0 | 0 | 2 | 5 |
| **714.81, Rheumatoid lung disease** | 1 | 0 | 2 | 0 | 0 | 1 | 1 | 1 | 0 | 7 | 13 |
| **Total** | **9** | **1** | **19** | **1** | **1** | **17** | **8** | **6** | **4** | **53** | **119**** |

*The total number of cases (N=119) differs from the total in Table 4 because this table does not include seven ICD-10-CM codes that did not have a previous ICD-9-CM diagnosis code.

**53 cases with no corresponding ICD-10-CM code: date of death prior to 10/01/2015 = 32; Medicare ABC enrollment ended prior to 10/01/2015 = 8; Diagnosis changed by the pulmonologist to a non-ILD code ≥after 09/30/2015 = 2; and No pulmonologist visit = 11
